# Parental Perceptions of Penicillin Allergy Labels: Findings from a Multisite Survey at Two Pediatric Primary Locations

**DOI:** 10.1017/ash.2025.259

**Published:** 2025-09-24

**Authors:** Elizabeth Monsees, Mary Lou Manning, Rana El Feghaly, Eileen Carter, Cliff O’Callahan, Sharon Hwang, Tara Schmidt, Monika Pogorzelska-Maziarz

**Affiliations:** 1 Children’s Mercy Hospital; 2 Thomas Jefferson University; 3 Children’s Mercy Kansas City; 4 University of Connecticut School of Nursing; 5 Middlesex Health and University of Connecticut; 6 Nemours Children’s Hospital; 7 Villanova University

## Abstract

**Background:** In children, penicillin allergy labels (PALs) are pervasive and persistent, despite linkage to suboptimal antibiotic selection with higher risk of side effects, increased length of hospitalization, and increased risk of harm throughout life. Up to 10% of children are labeled with PALs, yet over 95% tolerate the medication when tested. Parents might not always know that PALs are over-reported or incorrectly diagnosed. We aimed to examine parent and guardian perceptions of PALs and their attitudes towards delabeling. **Method:** We invited all English and Spanish-speaking parents of children presenting to two pediatric primary care locations in the northeast U.S to participate in an online, investigator-developed survey. Survey recruitment was passive, with parents discovering the survey through English and Spanish posters in the waiting and examination rooms. The survey included an initial screening question to identify whether a penicillin allergy was present. If the parent answered “yes,” they were instructed to proceed with survey completion. The survey consisted of 32 questions (7 reaction history, 9 perceptions, 5 provider interaction, 4 general knowledge, 6 demographics and one open-ended). We used descriptive statistics to analyze the data. **Result:** After screening, we received 54 completed responses. Most respondents had a college degree or higher (75%). When asked about the reaction, the majority occurred in those ≤ 2 years of life (55%); the predominant symptom reported was rash (92%). Twenty-nine percent of patients were evaluated in an urgent care or emergency room. Parents reported being very concerned by the reaction to penicillin (79%). When asked if their child would have a reaction if re-prescribed penicillin, none disagreed. Only 38% did not think allergies were permanent. Most families had not been offered penicillin testing (82%), although 67% expressed interest in the testing process, and 64% planned to inquire about testing following our survey. The majority (89%) would not agree to removing PALs without testing, citing fear that the child would have an allergic reaction if given penicillin (60%) and needing more information (25%) as the reasons for lack of agreement with PAL removal without testing. **Conclusion:** Among this highly educated population, parents expressed concerns at the initial reaction, perceived the reaction would reoccur with future penicillin use, and stated interest in testing, but were reluctant to delabel from history alone. Parents are untapped partners in delabeling; interventions are necessary to enhance parental understanding of the impact of PALs and the potential for delabeling with low-risk allergies.

